# MiR‐363‐3p promotes prostate cancer tumor progression by targeting Dickkopf 3

**DOI:** 10.1002/jcla.24360

**Published:** 2022-03-18

**Authors:** Li‐zhe Xu, Jin‐zhuo Ning, Yuan Ruan, Fan Cheng

**Affiliations:** ^1^ 117921 Department of Urology Renmin Hospital of Wuhan University Wuhan China

**Keywords:** apoptosis, DKK‐3, invasion, migration, miR‐363‐3p, proliferation, prostate cancer

## Abstract

**Background:**

Prostate cancer (PCa) is a frequent malignant tumor worldwide with high morbidity along with mortality. MicroRNAs (miRNAs) have been identified as key posttranscriptional modulators in diverse cancers. Herein, we purposed to explore the impacts of miR‐363‐3p on PCa growth, migration, infiltration along with apoptosis.

**Methods:**

The expressions of miR‐363‐3p along with Dickkopf 3 (DKK3) were assessed in clinical PCa specimens. We adopted the PCa cell line PC3 and transfected it using miR‐363‐3p repressors or mimic. The relationship of miR‐363‐3p with DKK3 was verified by a luciferase enzyme reporter assay. Cell viability along with apoptosis were determined by MTT assay coupled with flow cytometry analysis. Cell migration along infiltration were detected via wound healing, as well as Transwell assays. The contents of DKK3, E‐cadherin, vimentin along with N‐cadherin were analyzed via Western blotting accompanied with qRT–PCR.

**Results:**

MiR‐363‐3p was found to be inversely associated with the content of DKK3 in clinical PCa specimens. Further investigations revealed that DKK3 was miR‐363‐3p's direct target. Besides, overexpression of miR‐363‐3p decreased the contents of DKK3, promoted cell viability, migration coupled with infiltration, and reduced cell apoptosis, while silencing of miR‐363‐3p resulted in opposite influence. Upregulation of miR‐363‐3p diminished E‐cadherin contents but increased vimentin along with N‐cadherin protein contents in PC3 cells; in contrast, miR‐363‐3p downregulation produced the opposite result.

**Conclusion:**

Our study indicates that miR‐363‐3p promotes PCa growth, migration coupled with invasion while dampening apoptosis by targeting DKK3.

## Graphical abstract

MiR‐363‐3p was found to be negatively associated with the expression of DKK3 in clinical PCa specimens. Further studies revealed that DKK3 was a direct target of miR‐363‐3p. Moreover, overexpression of miR‐363‐3p downregulated the expression of DKK3, promoted cell viability, migration and invasion, and reduced cell apoptosis, while knockdown of miR‐363‐3p led to the opposite result. Upregulation of miR‐363‐3p decreased E‐cadherin levels but increased vimentin and N‐cadherin protein levels in PC3 cells; in contrast, miR‐363‐3p downregulation produced the opposite result.

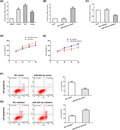

## 1 INTRODUCTION

Prostate cancer (PCa) is among the most frequent cancers in men with an estimated prevalence of 191,930 and 33,330 deaths in the United States in 2020.^1^, ^2^ Recently, the prevalence of PCa in China has rapidly increased annually. The usage of prostate‐specific antigen (PSA) has greatly helped us to distinguish PCa. However, PSA has a serious drawback, whereas its lack of specificity often results in overdiagnosis. This results in numerous unnecessary along with recurrent prostate biopsies, with the linked risks and overtreatment of clinically inconsiderable cancers.^3^, ^4^, ^5^ Although the current treatments for local PCa, which include surgical prostatectomy, chemotherapy, androgen deprivation therapy (ADT), immune therapy, radiation therapy, and improve the survival rate, severe side events remain. In contrast, advanced prostate cancer has remained as an incurable disease.^6^ Therefore, developing diagnostic, therapeutic, and prognostic hallmarks is a priority.

As a divergent Dickkopf (DKK) family member, DKK‐3 (Dickkopf‐related protein 3) is a tumor repressor implicated in slowing the progression of diverse kinds of cancer. DKK’s expression is lower in cancer cells, consisting of hepatocellular carcinoma, seminoma, renal clear cell carcinoma, PCa, cervical squamous carcinoma, along with non‐small‐cell lung cancer.^7^, ^8^ DKK3 primarily binds with LRP5/6 and acts as an antagonist of the Wnt/β‐catenin signaling pathway.^9^ Xu J et al.^10^ demonstrated that Exogenous DKK3 inhibited Wnt/β‐catenin signaling and cell proliferation in kidney cancer cells. It is also reported that DKK3 inhibits other Wnt transduction pathways such as the Wnt/JNK signaling pathway in tumor cells.^11^ Meanwhile, in many types of cancers, the failure of its normal expression is closely associated with CpG island methylation on the DKK3 promoter.^12^ Furthermore, a recent research investigation has documented that activation of DKK3 can dampen prostate cancer development.^13^ These results collectively illustrate that DKK3 plays an indispensable role in the progress of PCa.

MicroRNAs (miRNAs) constitute small endogenous noncoding molecules 19–24 nt long and modulate gene expression via degrading messenger RNAs (mRNAs), dampening protein biosynthesis or cross‐talking with long noncoding RNAs.^14^, ^15^ Accumulating evidence has confirmed that miRNAs participate in diverse biological processes, consisting of cell proliferation, apoptosis, differentiation and carcinogenesis. MiRNAs might also either repress tumor growth or enhance oncogenesis along with tumor progress.^16^, ^17^, ^18^ For instance, miRNA‐92a contents are lower in PCa cells, as well as dampen PCa cell viability along with metastasis through targeting SOX4.^19^ miR‐495 promotes bladder cancer cell growth as well as infiltration via dampening PTEN,^20^ and miR‐489‐3p dampens the growth, migration, coupled with infiltration of PCa cells but enhances apoptosis.^21^ Nonetheless, the biological effects of miR‐363‐3p during PCa progression and its underlying mechanisms have not been described.

Herein, we observed an inverse relationship of the content of miR‐363‐3p with DKK3 in PCa tumor tissues and exhibited DKK3 as a downstream direct target of miR‐363‐3p. Besides, we verified the modulatory effects of miR‐363‐3p on the growth, migration, infiltration, and apoptosis of PCa cells.

## 2 MATERIALS AND METHODS

### 2.1 Clinical tissues

This research premise was granted approval by the Clinical Research Ethics Committees of Renmin Hospital of Wuhan University (China) and conducted as per the guidelines of ethical management. A total of 24 PCa tissues and matching nonmalignant specimens were acquired from the Department of Urology, Renmin Hospital of Wuhan University from 2019–2020. All patients signed a form consenting to the utilization of their tissues and clinical information in this research. Stage and grade were established using World Health Organization classification systems and tumor node metastases (TNM). All tissues were split into two groups, with one‐half fixed using 4% PFA while the other was frozen immediately and stored at −80°C until analysis.

### 2.2 Cell lines and culture

LNCaP, PC3, and DU145 cells and the human nonmalignant prostate epithelium cell line RWPE1 were utilized in this research premise. The cell lines were acquired from the ATCC (American Type Culture Collection, Manassas, United States). These cell lines were inoculated into RPMI‐1640 medium (Gibco, United States) augmented with 10% FBS (Gibco, United States) under 5% CO_2_ and 95% O_2_ conditions.

### 2.3 Cell transfection

The cells were propagated with miR‐363‐3p repressors, mimic of miR‐363‐3p, or miR‐negative controls (NCs) via the Lipofectamine™ 2000 platform (Invitrogen) as per the manufacturers’ protocol. Posttransfection, the cells were incubated for 48 h before further treatment.

### 2.4 Plasmid creation along with luciferase enzyme reporter assays

The putative along with the mutated miR‐363‐3p target docking sequences in DKK3 were synthesized and propagated into a luciferase enzyme reporter to create a KKK‐3 WT (wild‐type) or DKK3‐MUT (mutated‐type) reporter plasmids. The wild‐type human DKK3 3’UTR fragment harboring predicted miR‐363‐3p target sites was amplified from PC3 cells using PCR. Overlap‐extension PCR was used to generate the mutant 3’UTR sequence of DKK3. Afterwards, wild‐type along with mutant 3′UTRs were sub‐propagated into a psiCHECK‐2 luciferase vector (Promega). In the luciferase enzyme reporter experiments, PC3 cells were planted onto 24‐well culture plates and co‐transfected with an miR‐363‐3p repressor or NC repressor via the Lipofectamine^®^ 2000 system. We harvested the cell transfects post transfection for 48 h. The activity of luciferase enzyme was assessed via the Dual‐Luciferase enzyme reporter assay platform (Promega).

### 2.5 Immunohistochemical staining

For this procedure, we fixed the tissues (in 4% PFA), dehydrated, and then paraffin‐embedment and segmentation at 4 μm thickness were done. Next, the segments were overnight inoculated with rabbit polyclonal anti‐DKK3 antibodies (Abcam, UK; ab187532) at 4°C. After washing three times with PBS, all segments were inoculated at room temperature with goat anti‐rabbit IgG for 30 min. Staining was then visualized with DAB and visualization done with the Olympus BX50 light microscope (Olympus Corporation).

### 2.6 Western blotting

RIPA lysis buffer with PMSF (Beyotime) was adopted to purify total protein. Afterwards, protein quantitation was done with the BCA (Beyotime) kit. In brief, fractionation of equivalent protein amounts was done on SDS–PAGE gels (10%) and further transferred onto PVDF membranes. Following the blocking, the membranes were inoculated overnight with primary antibodies against DKK3 (Abcam; ab187532), N‐cadherin (Abcam; ab18203), E‐cadherin (Abcam; ab1416), GAPDH and vimentin (Abcam, UK; ab92547) at 4°C. After that, they were inoculated with secondary antibodies for an hour, analysis was completed via an ECL (enhanced chemiluminescence) system kit.

### 2.7 RNA isolation and RT–qPCR

RNA isolation was done from the clinical specimens, as well as PCa cells with the TRIzol^®^ reagent (Invitrogen). Afterwards, cDNA was generated from the RNA with the Takara RNA PCR kit (Takara Biotechnology). Next, qPCR was conducted on the ABI 7900 Real‐Time PCR platform by utilizing an Applied Biosystems SYBR Green mix kit (Applied Biosystems Life Technologies). Relative miR‐363‐3p or DKK3 mRNA expression was normalized to U6 (for miRNAs) or GAPDH (for mRNAs), respectively. miRNA or mRNA relative amounts of were computed with the 2^−∆∆Ct^ approach. Oligonucleotide sequences are given in Table 1.

**TABLE 1:** RT–PCR primer sequences

GENE	Primer sequence (5′‐3′)
DKK3	F: CTGTGTGTCTGGGGTCACTG R: GCTCTAGCTCCCAGGTGATG
miR−363‐3p	F: AATTGCACGGTATCCATCTGTA R: CTCAACTGGTGTCGTGGA
U6	F: ATACAGAGAAAGTTAGCACGG R: GGAATGCTTCAAAGAGTTGTG
GAPDH	F: TCATTTCCTGGTATGACAACGA R: GTCTTACTCCTTGGAGGCC

### 2.8 Cell proliferation assay

To assess the influences of miR‐363‐3p on the cell viability, an MTT assay was used. 2 × 10^3^ PC3 cells/well were inoculated in 96‐well plates for 24, 48, 72, or 96 h. After treatment, we introduced 10 μl of MTT (5 mg/ml) to every well, and the plate was inoculated for 4 h at 37°C. After that we discarded the growth medium, and introduced 150 μl DMSO to disperse the formazan crystals. The absorbance was assessed on the ELX 800 microplate reader (Bio‐Tek Instruments) at 490 nm.

### 2.9 Cell apoptosis assay

The FITC annexin V apoptosis detection kit was utilized to perform apoptosis assays as described by the manufacturer (BD Pharmingen). In brief, we inoculated 10^5^ PC3 cells/mL in 6‐well plates. Thereafter, we incubated the cells in the dark with annexin V‐FITC (5 ml) along with PI (5 ml) for 15 min at RT prior to flow cytometry analysis (BD LSRII).

### 2.10 Wound healing assay

PC3 cells were inoculated in 6‐well plates in complete medium at 37°C for 12 h. Wounds were produced by using sterile 100 μl plastic pipette tips to generate gaps in the confluent cell layers. At 0 and 24 h after scratch creation, the wounds were assessed, and the gap distances of the migrating cells were determined with a microscope (Olympus).

### 2.11 Cell infiltration and migration assays

The migration and invasion potential of PC3 cells were explored with the Transwell chamber (Corning Life Sciences). We inoculated 1 × 10^5^ cells in serum‐free medium in the upper Matrigel‐coated compartment, and medium enriched with 10% FBS was introduced into the lower compartment. Following incubation at 37°C for 24 h, cells in the upper compartment membrane were wiped away. After that, the cells on the lower compartment were fixed, followed by staining in 0.1% crystal violet for half an hour, followed by imaging.

### 2.12 Statistical analysis

All data are given as the means ± SD. Statistical differences were evaluated with a two‐tailed Student's *t* test or χ^2^ test as indicated. *p* < 0.05 indicated statistical significance. Every experiment was repeated at least in triplicate. The statistical analyses were implemented in SPSS v2.0.

## 3 RESULTS

### 3.1 The content of miR‐363‐3p inversely correlates with the contents of DKK3 in PCa samples

**FIGURE 1:**
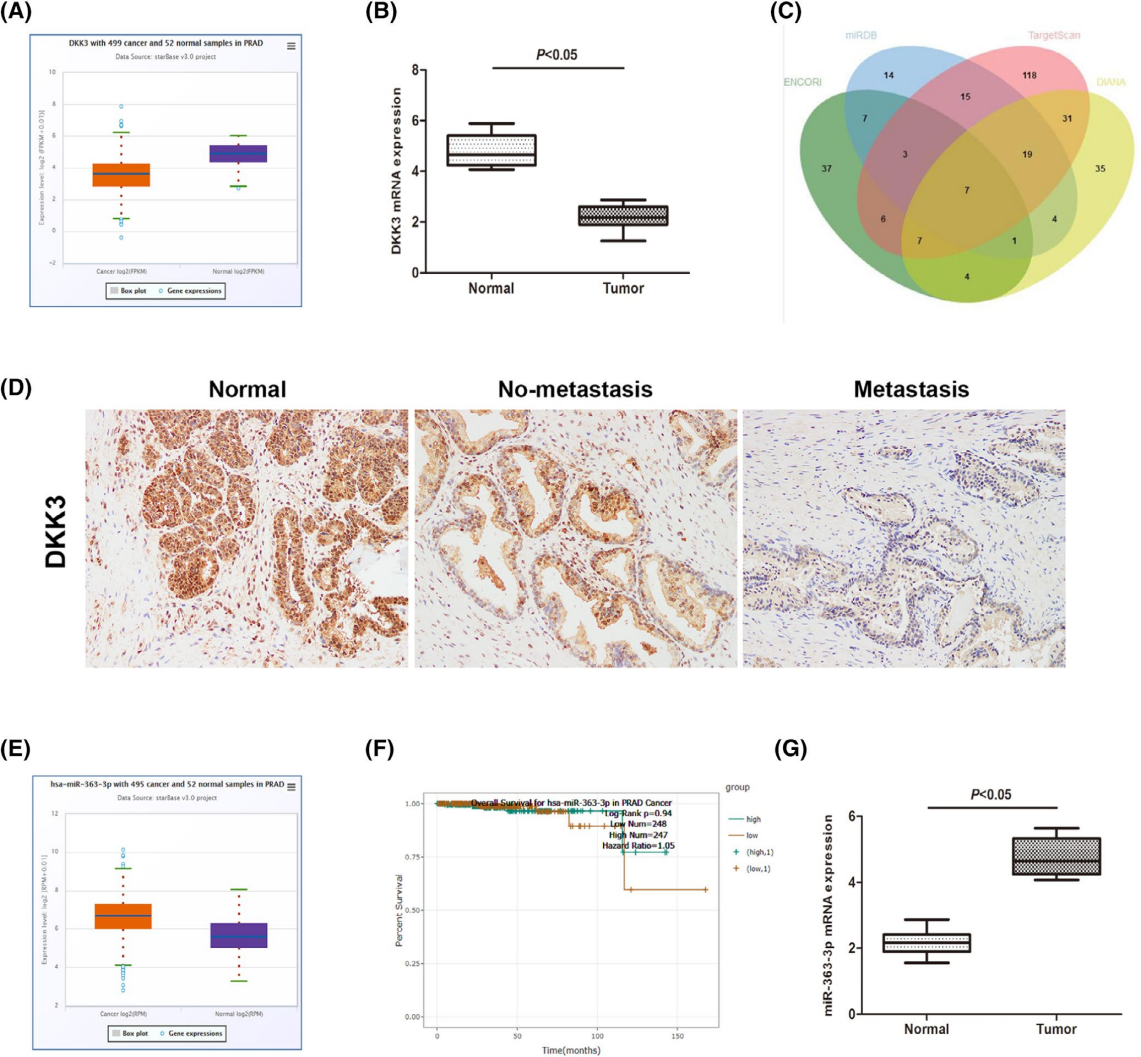
The expression of miR‐363‐3p is inversely linked to the expression of DKK3 in PCa samples. (A), Data from starBase 3.0 illustrated that DKK3 was remarkably decreased in PCa tissues in contrast with nonmalignant tissues. (B) The qRT–PCR results illustrated that DKK3 was remarkably downregulated in the PCa group in contrast with the nonmalignant group. (C) A Venn diagram was used to select the intersection of the potential regulatory miRNAs of DKK3 predicted by the four databases. (D) Immunohistochemical staining of DKK3 in nonmalignant prostate tissues, nonmetastatic PCa tissues and metastatic PCa tissues. (E) The database illustrated that miR‐363‐3p was remarkably increased in PCa tissues in contrast with nonmalignant tissues. (F) Data from starBase 3.0 illustrated that patients harboring low expression of DKK3 had a poorer OS time than patients harboring high‐DKK3 expression. (G) The qRT–PCR data illustrated that miR‐363‐3p was remarkably upregulated in the PCa group in contrast with the nonmalignant group. Data are given as the means ± SD of three independent experiments (PCa, prostate cancer; *p < 0.05 Respective NC group)

To explore the expression of DKK3 in PCa and nonmalignant tissues, we assessed the content of DKK3 in the TCGA data resource from starBase V3.0. As depicted in Figure 1A, DKK3 expression was remarkably downregulated in PCa tissues in contrast with nonmalignant tissues. Then, we analyzed 24 PCa tissue specimens matched to their adjacent nonmalignant tissues to determine the expression of DKK3 by qRT–PCR and immunohistochemical staining (Figure 1B, D). The expression levels of DKK3 were markedly lower in PCa tissues than in neighboring nonmalignant tissues. These results were consistent with those from the database. Additionally, we performed bioinformatics analysis (using TargetScan,^22^ miRDB,^23^ DIANA^24^ and ENCORI^25^) to assess possible regulatory miRNAs of DKK3. A total of seven overlapped miRNAs were screened for the following investigations, including miR‐363‐3p, miR‐25‐3p, miR‐32‐5p, miR‐92a‐3p, miR‐92b‐3p, miR‐3126‐5p, and miR‐664b‐3p. MiR‐363‐3p was obtained from the common prediction of four databases (Figure 1C). TCGA data from the starBase V3.0 platform revealed elevated content of miR‐363‐3p in PCa samples in contrast with that in matching nonmalignant samples (Figure 1E). Moreover, the database exhibited that the high‐miR‐363‐3p expression patients had an obviously poorer OS time in contrast with those with low miR‐363‐3p expression (Figure 1F). Thereafter, we validated this result using tissues from our department in the same way (Figure 1G).

### 3.2 MiRNA‐363‐3p modulates the growth, as well as promotes the apoptosis of PCa cells

**FIGURE 2:**
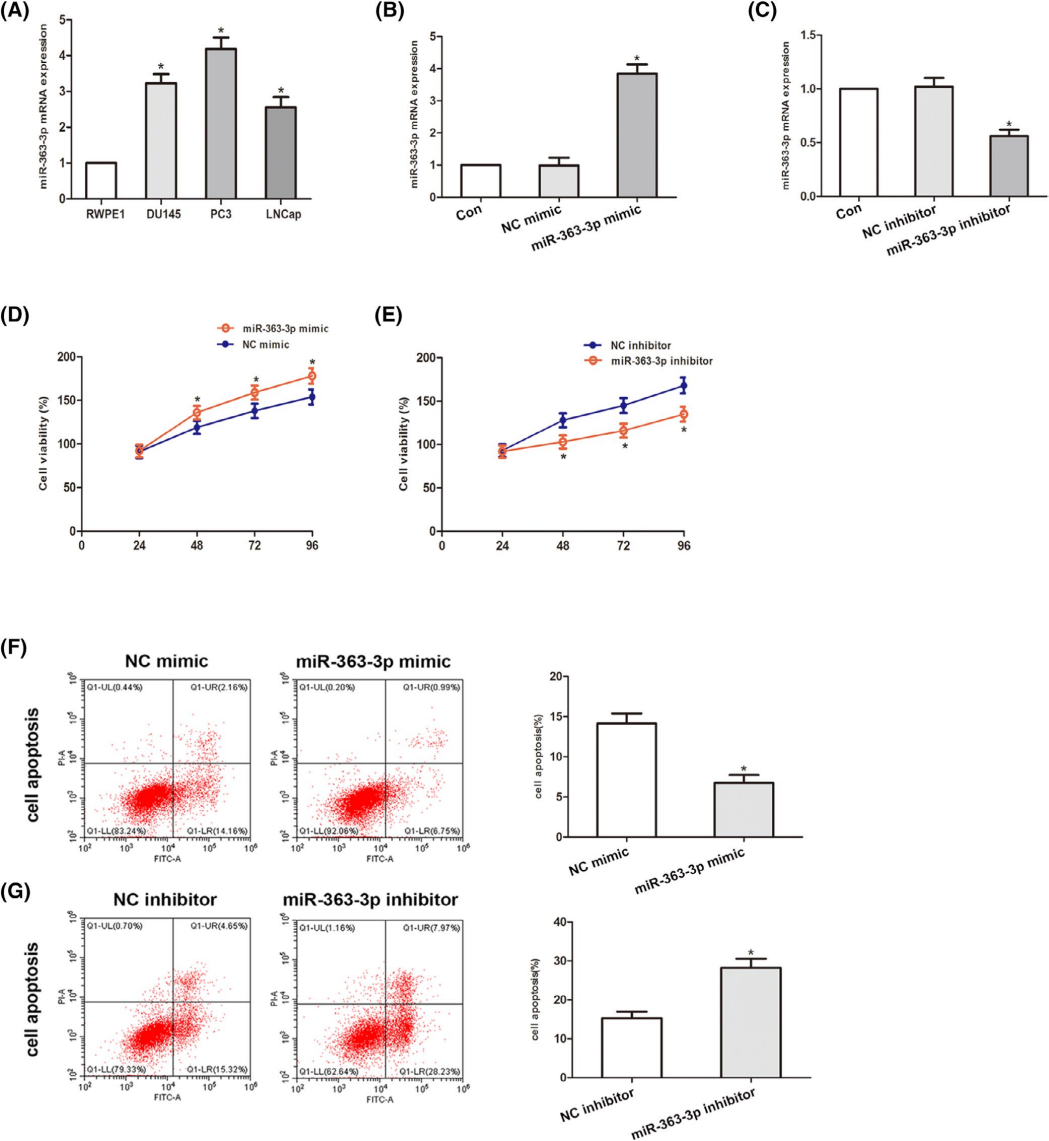
MiR‐363‐3p modulates the proliferation along with apoptosis of a PCa cell line. (A) MiR‐363‐3p content in the DU145, PC3, LNCaP, and RWPE1 cell lines. (B and C) miR‐363‐3p content was examined in PC3 cells with miR‐363‐3p repressors and mimic via qRT–PCR. (D and E) An MTT assay to explore cell viability in cell transfects of miR‐363‐3p mimic and miR‐363‐3p repressors. The absorbance values were determined at 24, 48, 72, and 96 h posttransfection. (F and G) Flow cytometry was conducted 48 h posttransfection. The apoptotic cell rate is shown in the histogram. Data are given as the means ± SD of three independent experiments (*p < 0.05 Respective NC group)

To further ascertain the relationship of miR‐363‐3p with DKK3, we first explored the content of miR‐363‐3p in PCa cell lines (LNCap, DU145, and PC3) and a RWPE‑1 human prostate epithelial cell line. The data from qRT–PCR analysis exhibited that miR‐363‐3p content was elevated to varying degrees in PCa cells in contrast with nonmalignant cells (Figure 2A). As PC3 cells exhibited the greatest mean miR‐363‐3p expression in contrast with RWPE‐1 cells, they were selected for all subsequent experiments. We transfected PC3 cells with miR‐363‐3p mimic or repressors and then obtained miR‐363‐3p‐overexpressing or miR‐363‐3p‐silenced cells (Figure 2B, C). To elucidate the role of miR‐363‐3p in the growth of PCa cells, an MTT assay illustrated that the viability of PC3 cell transfects of miR‐363‐3p mimic was remarkably promoted relative to the controls, cell transfects of miR‐363‐3p inhibitor exhibited remarkably dampened cell viability (Figure 2D, E). In addition, we assessed the role of miR‐363‐3p on PCa cell apoptosis. The flow cytometry data illustrated that overexpression of miR‐363‐3p resulted in a remarkably lower percentage of apoptotic nuclei, and this inhibition could be reverted by the usage of miR‐363‐3p inhibitor (Figure 2F, G). Collectively, the above‐mentioned results illustrate that miR‐363‐3p may modulate the growth along with apoptosis of PCa cells.

### 3.3 MiR‐363‐3p promotes the migration and infiltration of PCa cells

**FIGURE 3:**
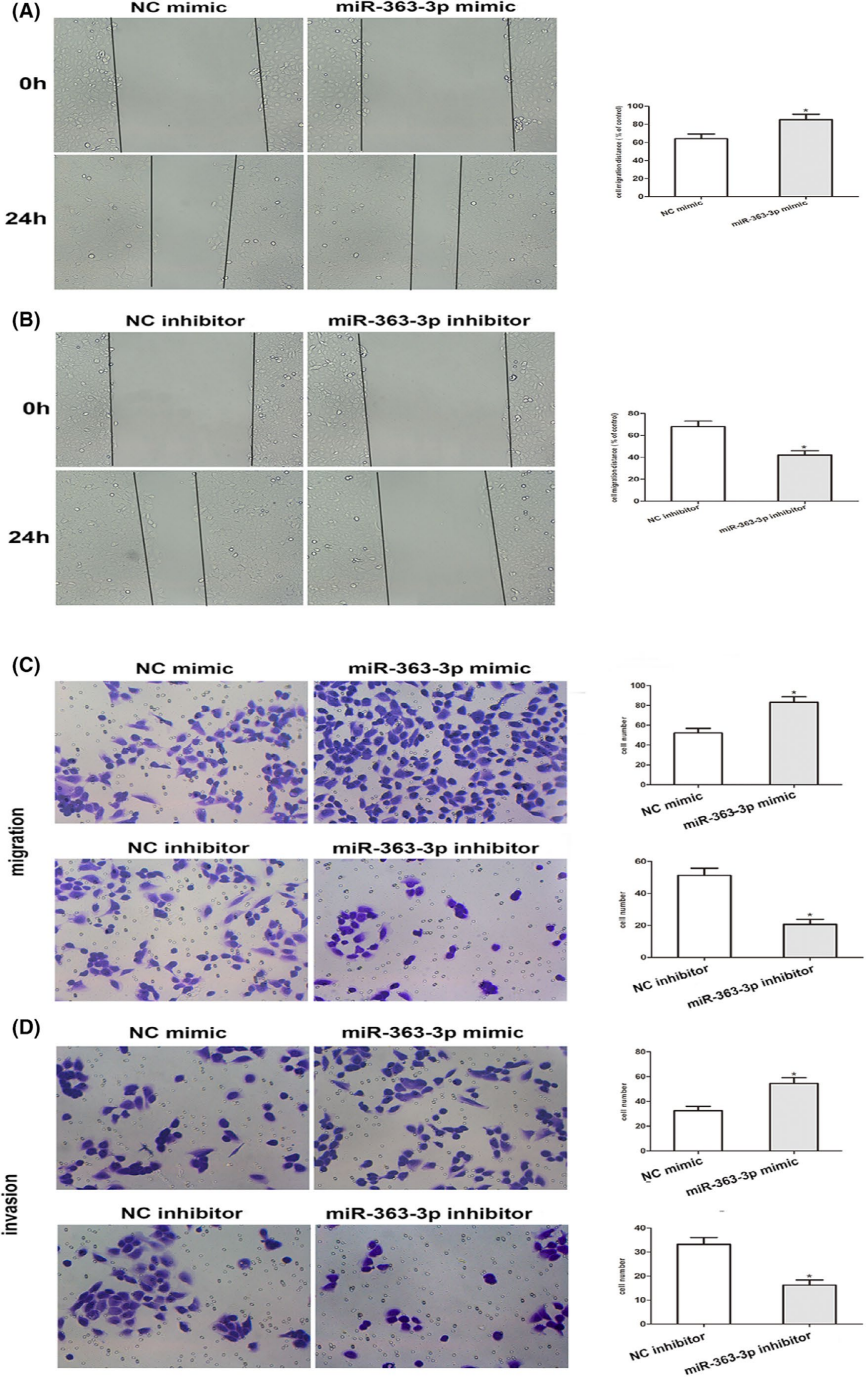
MiR‐363‐3p promotes the migration along with infiltration of PCa cells. (A and B) A wound healing assay was carried out to explore the migratory potential of PC3 cell tansfects miR‐363‐3p repressors or miR‐363‐3p mimic after 0 and 24 h. © Transwell migration assays were conducted to assess the migratory ability of PC3 cell transfects of miR‐363‐3p mimic or miR‐363‐3p inhibitor. (D) Transwell infiltration assays using Matrigel‐coated membranes were performed to show the infiltrative abilities of PC3 cell transfects of miR‐363‐3p repressors or miR‐363‐3p mimic (*p < 0.05 vs. Respective NC group)

Next, we investigated the effects of miR‐363‐3p on the migratory along with infiltrative potential of PCa cells. The wound healing assay exhibited that silencing of miR‐363‐3p dramatically diminished PC3 cell migration, while overexpression of miR‐363‐3p decreased PC3 cell migration (Figure 3A, B). Through the Transwell assay, we established that upregulation of miR‐363‐3p facilitated cell migration along with infiltration, while downregulating miR‐363‐3p dampened cell migration along with invasion (Figure 3C, D). Hence, these data strongly illustrated that miR‐363‐3p enhanced cell migration along with infiltration in PCa cells.

### 3.4 MiR‐363‐3p directly targets DKK3 and inversely modulates DKK3 expression

**FIGURE 4:**
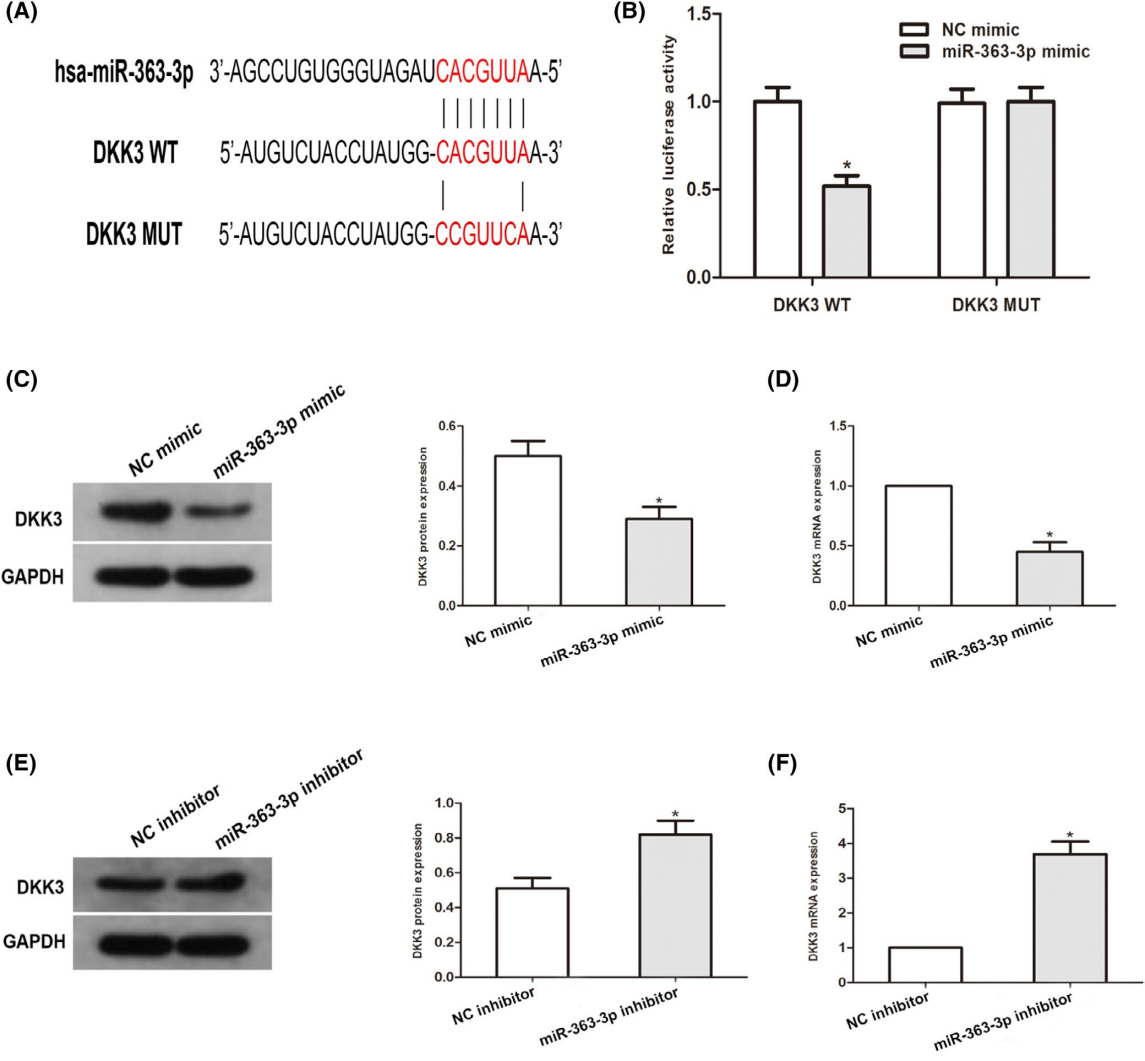
MiR‐363‐3p directly targets DKK3, then inversely modulates DKK3 expression. (A) Sequence alignment of the predicted miR‐363‐3p docking sites within the DKK3 3’UTR along with its mutated sequence for the luciferase enzyme reporter assay. (B) A luciferase enzyme reporter assay was conducted in PC3 cell co‐transfects of miR‐363‐3p mimic and reporter vectors harboring the mutated DKK3 3’UTR or DKK3 3’UTR. A comparison of the activities of luciferase enzyme are given. (C–F), Western blotting along with qRT–PCR assessment of DKK3 expression in cell transfects of miR‐363‐3p repressors or miR‐363‐3p mimic in PC3 cells. (*p < 0.05 vs. Respective NC group)

We next predicted that miR‐363‐3p was an upstream modulator of DKK3 via the open access software (TargetScan, miRDB, DIANA, and ENCORI), as well as a putative docking site in the 3'UTR of DKK3 for miR‐363‐3p was established (Figure 4A). To validate this prediction, a luciferase enzyme reporter gene assay was carried out. The results illustrated that the luciferase activity of the DKK3 3'UTR reporter was dramatically diminished in miR‐363‐3p overexpressing cells. Nonetheless, this influence was terminated by mutation in the putative docking site within the 3′UTR of DKK3 (Figure 4B). By using Western blotting along with qRT–PCR analyses, we assessed the content of DKK3. The data illustrated that DKK3 content was remarkably downregulated in miR‐363‐3p‐overexpressing cells but moderately elevated in miR‐363‐3p‐silenced cells (Figure 4C–F). Overall, these findings illustrated that miR‐363‐3p directly repressed DKK3 expression through direct targeting.

### 3.5 Impacts of miR‐363‐3p on EMT (epithelial–mesenchymal transition) in PCa cells

**FIGURE 5:**
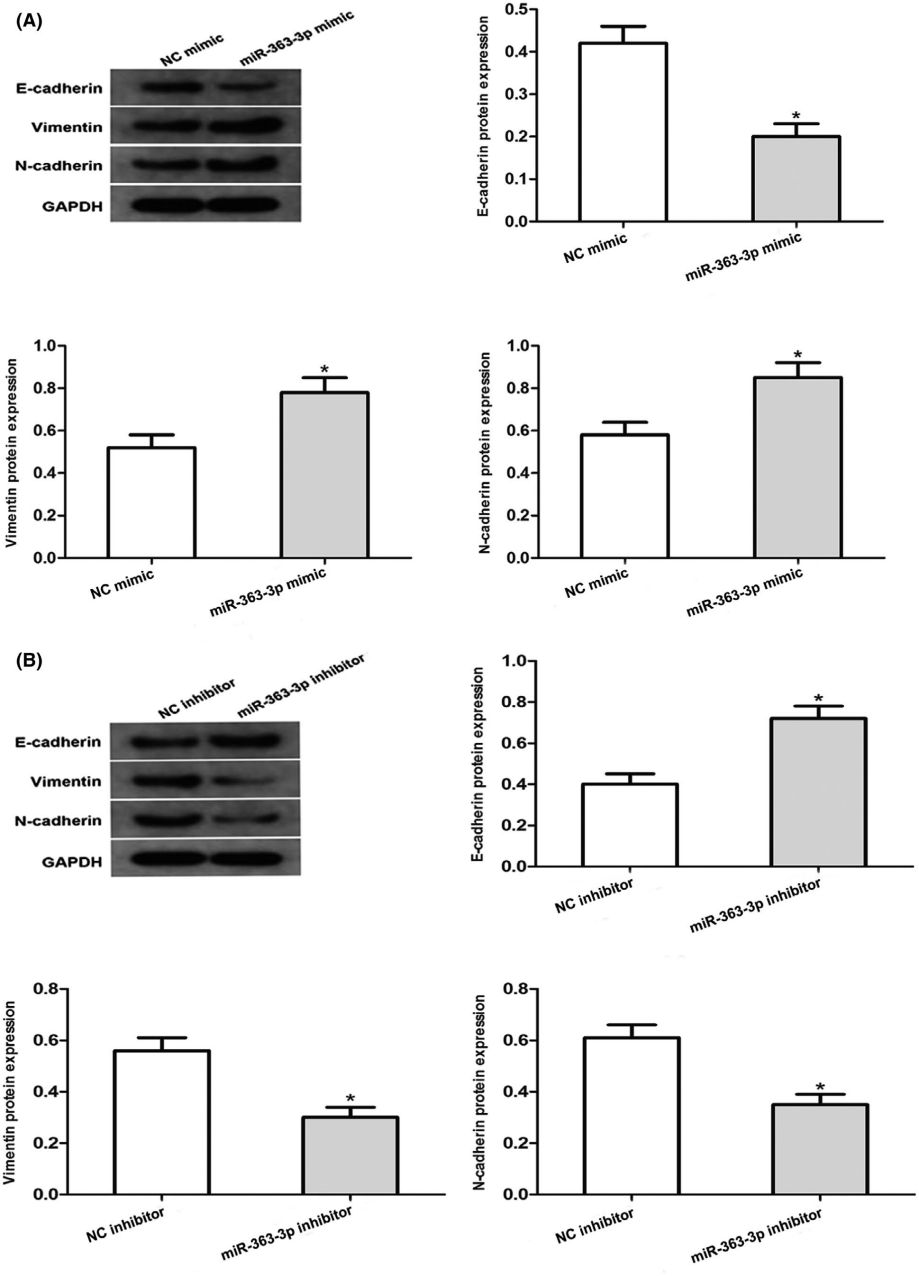
Impacts of miR‐363‐3p on EMT (epithelial–mesenchymal transition) in PCa cells. E‐Cadherin, vimentin as well as N‐cadherin protein contents in PC3 cell transfects of miR‐363‐3p repressors or miR‐363‐3p mimic were assayed via Western blotting. GAPDH served as the standardization control (*p < 0.05 vs. Respective NC group)

To understand the impact of miR‐363‐3p on the EMT progress of PCa, we transfected miR‐363‐3p repressors or mimic into PC3 cells. Western blotting data illustrated that miR‐363‐3p overexpression diminished E‐cadherin content but elevated vimentin along with N‐cadherin protein contents in PC3 cells (Figure 5A). On the contrary, the miR‐363‐3p inhibitor elevated the contents of E‐cadherin and diminished those of vimentin along with N‐cadherin in PC3 cells (Figure 5B). Hence, these data illustrated that miR‐363‐3p may work as a key modulator of the EMT progress of PCa.

## 4 DISCUSSION

PCa is the most often diagnosed solid tumor among men and the second primary cause of cancer‐linked death in men in developed countries, as an estimated 3 million men in the USA are living with this disease.^26^, ^27^ Apart from surgery along with radiotherapy, ADT (androgen deprivation therapy) is the standard treatment of advanced prostate cancer. Commonly used therapeutic agents include enzalutamide, bicalutamide and abiraterone.^28^ Although there have been numerous advances in diagnostic and therapeutic regimens, the long‐term prognosis or 5‐year OS (overall survival) of PCa patients is still relatively poor.^29^ Therefore, to improve treatment for PCa patients, it is very remarkable to explore effective biomarkers and the potential molecular mechanisms in PCa.^30^

It has been documented that DKK3 is abnormally expressed in numerous kinds of cancer and can suppress cancer cell migration, invasion, and proliferation through multiple pathways.^31^, ^32^ Moreover, recent studies have confirmed an association between DKK3 and PCa, and DKK3 can suppress tumor progression and work as a tumor repressor gene in PCa cells.^13^, ^34^ We identified a docking correlation of miR‐363‐3p with DKK3 through open access bioinformatic data resources. Hence, we chose miR‐363‐3p as the key miRNA for further study. Herein, we confirmed that DKK3 was inversely correlated with miR‐363‐3p in prostate cancer specimens. The miR‐363‐3p level was remarkably increased in PCa tissues in contrast with paired nonmalignant tissues, while DKK3 was remarkably downregulated in tumors. Subsequently, we utilized the luciferase enzyme reporter assay to identify that DKK3 is a direct downstream target of miR‐363‐3p. These data illustrated that the expression levels of DKK3 are inversely modulated via miR‐363‐3p in PCa cells, which is congruent with the data of clinical specimens.

Diverse investigations have illustrated that miRNAs participate in numerous biological processes, for instance cell growth, apoptosis, as well as differentiation.^34^ It has been documented that miR‐363‐3p is expressed aberrantly in various cancers, for instance glioma, liver cancer, along with non‐small‐cell lung cancer.^36^, ^37^, ^38^ Furthermore, a recent investigation exhibited that miR‐363‐3p suppressed tumor progression by directly targeting SOX4 in osteosarcoma.^38^ Nonetheless, the clinical significance coupled with the biological roles of miR‐363‐3p in the progress of PCa are not fully understood. In our in vitro studies, we utilized PC3 cells to trigger the progress of PCa. The data illustrated that silencing of miR‐363‐3p dampened cell migration, infiltration and growth and suppressed apoptosis in PCa cell lines, while miR‐363‐3p overexpression led to the opposite influences. In summary, our results suggest that miR‐363‐3p works on an oncogene and has the potential to be a good prognostic biomarker and treatment target in PCa.

Tumor metastasis constitutes the primary cause of cancer mortality, and is a multistep process involving attachment to, degradation of, as well as detachment from an ECM, and lastly, active migration far from the primary tumor.^39^ EMT is a remarkable morphogenetic event in PCa cell infiltration coupled with metastasis from primary tumors and is typified via downregulation of E‐cadherin, along with upregulation of N‐cadherin as well as vimentin.^40^ Our observations indicate that knockdown of miR‐363‐3p caused a remarkable increase in E‐cadherin coupled with a reduction in N‐cadherin along with vimentin levels, whereas miR‐363‐3p overexpression caused the opposite result, illustrating that miR‐363‐3p‐triggered dampening of DKK3 was correlated with EMT during the progress of PCa.

## 5 CONCLUSION

Our data indicate that miR‐363‐3p might play an indispensable role in the tumorigenesis of PCa cells by promoting cell growth, migration along with infiltration and restraining cell apoptosis, which may be ascribed to the inverse modulation of DKK3, at least in part.

## CONFLICT OF INTEREST

The authors declare that they have no conflict of interest.

## CONSENT FOR PUBLICATION

All patients signed consent for publication.

## Data Availability

The datasets used and analyzed during the current study are available from the corresponding author on reasonable request.
